# Prey identity affects fitness of a generalist consumer in a brown food web

**DOI:** 10.1002/ece3.9207

**Published:** 2022-08-23

**Authors:** Lily Khadempour, Leslie Rivas Quijano, Casey P. terHorst

**Affiliations:** ^1^ Department of Biology California State University, Northridge Northridge California USA; ^2^ Present address: Department of Earth and Environmental Sciences Rutgers University Newark New Jersey USA

**Keywords:** bottom‐up effects, phytotelmic, predator–prey dynamics, *Sarracenia purpurea*

## Abstract

The use of ever‐advancing sequencing technologies has revealed incredible biodiversity at the microbial scale, and yet we know little about the ecological interactions in these communities. For example, in the phytotelmic community found in the purple pitcher plant, *Sarracenia purpurea,* ecologists typically consider the bacteria as a functionally homogenous group. In this food web, bacteria decompose detritus and are consumed by protozoa that are considered generalist consumers. Here, we tested whether a generalist consumer benefits from all bacteria equally. We isolated and identified 22 strains of bacteria, belonging to six genera, from *S. purpurea* plants. We grew the protozoa, *Tetrahymena* sp. with single isolates and strain mixtures of bacteria and measured *Tetrahymena* fitness. We found that different bacterial strains had different effects on protozoan fitness, both in isolation and in mixture. Our results demonstrate that not accounting for the composition of prey communities may affect the predicted outcome of predator–prey interactions.

## Graphical abstract

*Sarracenia purpurea*, the purple pitcher plant, is used as model system for studying ecology and evolution in a constrained natural community. The bacteria that serve as the base of this brown food web are often treated as a functionally similar as the food source for protozoa. Through our experiment, we demonstrate that bacterial strain identity matters for protozoan fitness and that this should be a consideration in future studies looking at bottom‐up effects in this system.

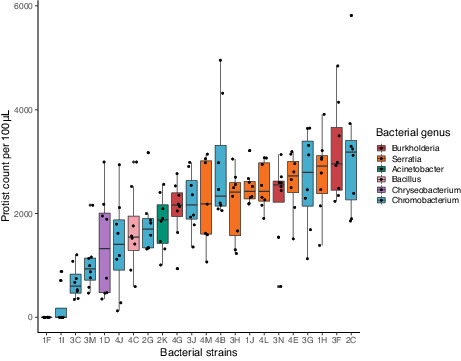

## 1 INTRODUCTION

Predator–prey or consumer‐resource dynamics are among the best studied ecological interactions. Theory suggests that the coexistence of predator and prey depends on several parameters that incorporate traits of both predator and prey, such as attack rate, handling time, and conversion efficiency (Holling, 1959). Considerable theory has explored how variability of predator and prey traits affects population dynamics, demography, and species coexistence (e.g., Abrams & Rowe, 1996; Fleischer et al., 2018; Kendall et al., 1999; Peckarsky et al., 2008). Many empirical examples demonstrate how different prey traits affect handling time (e.g., Faria et al., 2004; Werner, 1974) and attack rates of predators (Boates & Goss‐Custard, 1992; Elliott, 2003) ultimately having consequential effects on predator fitness. It would come as no surprise to many ecologists that the identity of an animal's prey affects performance and fitness, yet in the microbial world, we have far less understanding of the specificity of trophic interactions.

Most examples of trophic interactions emerge from green food webs, where photosynthetic plants or algae form the base of the food web. However, brown food webs, in which detritivores, such as bacteria and fungi, form the base of the food web, are also common in nature. In green food webs, ecologists are keenly aware of how traits of the plant community affect the transfer of energy through a food web (Mooney et al., 2010; van der Stap et al., 2007). However, in brown food webs, the species composition of the base of the food web has historically been treated as a black box where bacteria or fungi are treated as a single taxonomic unit (e.g., Miller & terHorst, 2012). Despite rapid advances in identifying microbial taxa in natural communities over the past decade, we still have far less knowledge of the ecological role of specific taxa and how they interact with other species. The effect of different bacterial species on consumer growth rates has remained largely untested (but see Darby & Herman, 2014; Mohapatra & Fukami, 2005), even though variation in bacterial species traits is likely to alter consumer attack rates, handling times, and conversion efficiencies.

The phytotelmic (organisms that inhabit small pools of water within or upon plants) community found in the leaves of the purple pitcher plant (*Sarracenia purpurea*) has been used as a model system for studying broad questions about ecology and evolution (Cochran‐Stafira & von Ende, 1998; Ellison & Gotelli, 2002; Kneitel & Miller, 2002; Miller et al., 1994, 2014). The pitcher‐shaped leaves attract insects and serve as pitfall traps in which insects drown, decompose, and provide nutrients to the plant. The insects serve as the source of energy and nutrients at the bottom of a brown food web. Bacteria decompose the dead insects and are consumed by a suite of protozoa and rotifer species, which are consumed by mosquito larvae. Numerous top‐down studies have demonstrated that the evolutionary and ecological dynamics of protozoan consumers affect bacterial community composition (Canter et al., 2018; Cochran‐Stafira & von Ende, 1998; Holdridge et al., 2016; Paisie et al., 2014; Peterson et al., 2008). These top‐down effects align with protozoan selective feeding behavior in other systems (Gaines et al., 2019; Strom & Loukos, 1998), which influences prey community dynamics. Selective feeding behavior may also be driven by bottom‐up forces, such as variability in prey quality, which in turn affect consumer fitness. There has been less work exploring the bottom‐up effects of different bacterial species on protozoan ecological dynamics. Although some studies have examined the effects of total bacterial abundance on higher trophic levels (e.g., Hoekman, 2007; Kneitel & Miller, 2002), most studies indirectly manipulate the bacterial community as a whole by altering resource availability, rather than particular taxa of bacteria with different traits. An underlying assumption with these studies is that the protozoa are generalist consumers of bacteria and that, regardless of their identity, a higher abundance of bacteria promotes protozoan growth.

Here, we examine this assumption and ask whether different strains of bacteria differentially affect consumer fitness. We collected fluid from *S. purpurea* pitcher plants found in the field, from which we isolated single strains of bacteria. We quantified the effects of single strains and multi‐strain communities of bacteria on the fitness of a common ciliate (*Tetrahymena* sp.), which is found in *S. purpurea* phytotelmic communities, and is commonly used in lab microcosm experiments in this system. We predict that different strains of bacteria will differentially affect protozoan fitness.

## 2 METHODS

### 2.1 Isolation of bacteria

We collected water contained within pitcher plant leaves haphazardly from leaves of various ages in one field in the Apalachicola National Forest in northern Florida (USA). Large insect parts were filtered from the fluid, and 2% v/v DMSO was added before freezing at −20°C in 50‐ml conical tubes. Samples were shipped to California State University, Northridge, where we thawed the tubes and diluted the liquid with sterilized water at 10×, 100×, and 1000× dilutions. We spread 50 *μ*l of liquid from each dilution onto LB solid media plates (Cold Spring Harbor Laboratory Press, 2016) using a plate spreader. We monitored these plates daily to check for bacterial colony growth and then identified different morphotypes of bacteria, which were picked and streaked in order to isolate individual strains. Once we were confident the bacteria were in isolation, both through visual confirmation and sequencing (see below), we maintained them in 5 ml of liquid LB media (Cold Spring Harbor Laboratory Press, 2016) and transferred them to fresh tubes every 2 weeks.

### 2.2 Identification of bacteria

We extracted DNA from bacterial strains using the Qiagen Blood and Tissue Kit, with the specifications for bacterial cultures. We used 16S rRNA gene primers 27F and 1392R in a PCR with the following conditions: 94°C for 5 min, 30 cycles of 94°C for 20 s, 55°C for 20 s, and 72°C for 70 s, with a final elongation of 72°C for 10 min. We sequenced the amplicons with Sanger sequencing through Laragen with both forward and reverse primers, then trimmed, identified, and analyzed sequences using 4Peaks and CLC Sequence Viewer 7. We used NCBI BLAST to identify the strains to the genus level using their 16S sequences. Ultimately, we isolated 22 individual unique strains of bacteria (Table S1).

### 2.3 Isolation of protozoa

For several years, we have maintained eight strains of the ciliated protozoa *Tetrahymena* sp. in lab cultures. These strains were originally collected from different pitcher plants in the Apalachicola National Forest and have been maintained independently in the laboratory. For this experiment, we isolated each of these strains from their associated bacterial community. We created YPD sterile media (YPD media, 2010) supplemented with four filter‐sterilized antibiotics (final concentration in parentheses): kanamycin (50 mg/ml), tetracycline (10 mg/ml), penicillin (1200 mg/ml), and streptomycin (120 mg/ml). We added 100 *μ*l of each *Tetrahymena* strain to separate replicate test tubes with media and allowed them to grow for 3 days at room temperature. We then looked for bacteria in a small volume of the protist culture at 1000× magnification. We only used replicate tubes in which little or no bacteria were visible. This technique was unlikely to have removed all bacteria, but the concentration of bacteria in these cultures was many orders of magnitude lower than the bacteria that we added in the experimental tubes below. We then added each protist strain to the experimental tubes, as described below.

### 2.4 Effect of individual bacterial strains

We established microcosms by adding 10 mg of crushed Tetracolor Fish Flakes to 10 ml of water in a test tube before autoclaving for 45 min at 120°C. We added 30 *μ*l of liquid culture of an individual bacterial strain to each tube and allowed these bacteria to establish and grow for 1 day at ambient room temperature. We did not control for bacterial abundance as we considered bacterial growth rate on the media as one of several bacterial traits that could potentially differ among strains. We then added an individual *Tetrahymena* strain to different tubes, so that each *Tetrahymena* strain was grown with each bacterial strain; each *Tetrahymena* strain served as a replicate (*n* = 8) in testing the effects of the bacterial strains. The initial density of protozoa in each tube was 100 individuals per ml. We allowed the bacteria and protozoa to grow together for 4 days at ambient room temperature and then counted the density of protozoa using Palmer cells (Wildlife Supply Company).

### 2.5 Effect of multiple bacterial strains

To determine whether different bacterial strains would have different effects when combined with other bacterial strains in a community context, we also created synthetic communities of bacteria. We were particularly interested in whether strains that positively affected protozoan fitness could compensate for strains that negatively affected protozoan fitness. This would reveal if the bad strains failed to provide enough nutrition (in which case we would expect good strains in a community to compensate) or if they were directly harming consumers, through toxin production for example (in which case, we would expect poor consumer performance even when other strains were present). We created five different combinations of six bacterial strains. Communities 1–4 all contained either one or both of strains 1F and 1I, the two worst strains for protozoan fitness, based on the first experiment. The other strains in the community were chosen haphazardly to create different communities of six strains. Community 5 was the only community to exclude the two worst strains 1F and 1I (Table S2). We grew each of these five bacterial communities with each of the eight unique *Tetrahymena* strains. We mixed equal volumes of the six strains in each community and then aliquoted 30 *μ*l of this mixture into each replicate experimental tube. We allowed these bacterial communities to grow for 1 day before adding protozoa and quantifying their growth in the same way as in the previous experiment.

For both experiments, we used ANOVA to examine the effects of bacterial strain or mixture on the final abundance of protozoa. *Tetrahymena* strains were considered as independent replicates. We followed significant treatment effects with Tukey's HSD to examine pairwise differences among treatment levels, using the “agricolae” package (de Mendiburu & Yaseen, 2020) in R 3.6.3 (R Core Team, 2013).

## 3 RESULTS AND DISCUSSION

**FIGURE 1:**
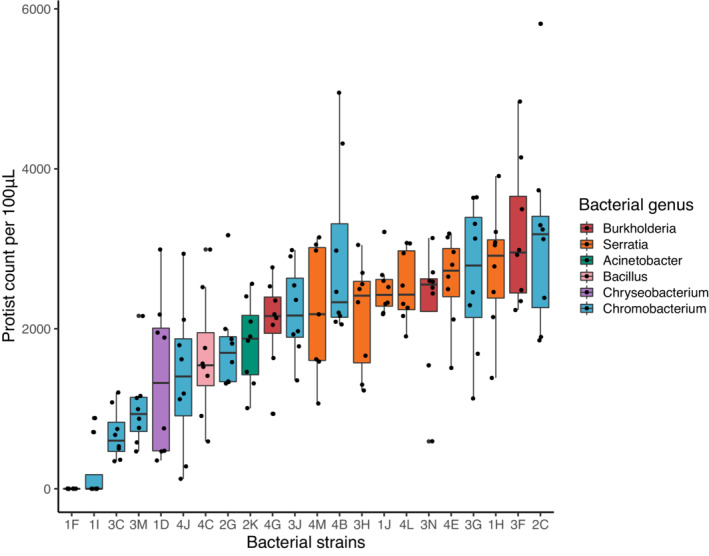
Protist abundance depends on the identity of bacterial strain (*F*_[21,153]_ = 14.23, *p* < .001). Box plot definitions: Center line—median, upper, and lower box limits—upper and lower quartiles, whiskers—1.5× inter‐quartile range, outliers—any points outside the 1.5× inter‐quartile range

In total, we isolated 22 bacterial strains with different morphotypes, belonging to six genera: *Serratia, Chromobacterium, Chryseobacterium, Burkholderia, Acinetobacter*, and *Bacillus*. Individual strains of bacteria had different effects on protozoan fitness (*F*_[21,153]_ = 14.2, *p* < .001; Figure 1). Some bacterial taxa resulted in highly abundant protozoan populations, although other taxa either could not support protozoan growth or actively inhibited it, and many other taxa had varying degrees of intermediate effect.

If *Tetrahymena* is indeed a generalist consumer with regard to what it consumes, then it is decidedly not a generalist with regard to the benefits it receives from consuming prey. Instead, bacterial taxa reside on a broad spectrum with respect to their effects on protozoan fitness. Although many studies in this system assume that protozoan fitness is primarily determined by the abundance of bacterial prey, our results suggest that fitness may be largely determined by the composition of the bacterial community.

Two *Chromobacterium* strains (1F and 1I) had especially negative effects on protozoan fitness, with nearly no protozoa observed in these cultures. This is consistent with previous studies that have shown that *Chromobacterium* spp. inhibit protozoan growth through the production of the pigment violacein (Pickup et al., 2007; Singh, 1945). For this reason, *Chromobacterium* has been targeted as a potential source of compounds for treating fungal and viral infections, as well as cancer cell growth (Cheng et al., 2007; Sasidharan et al., 2015). However, other strains of *Chromobacterium* (4B, 3G, and 2C) produced some of the highest protozoan abundances. This emphasizes that the ecological function of bacteria cannot be generalized, even among closely‐related taxa; single strains of bacteria may not well represent the effects of other species in the same genus. Other genera, such as *Burkholderia* and *Serratia*, had consistently positive effects on protozoan fitness, although more isolation and testing of other taxa within these genera are necessary to determine if this is generally true. The effects of bacterial traits on protozoan growth are not limited to only toxicity, as there were a range of intermediate effects of different bacterial taxa. Other factors such as, but not limited to, growth rate, motility, and nutritive quality might also affect protozoan fitness, and a more detailed analysis of bacterial traits and their effects on protozoans would provide for interesting future work (Goyal et al., 2021).

**FIGURE 2:**
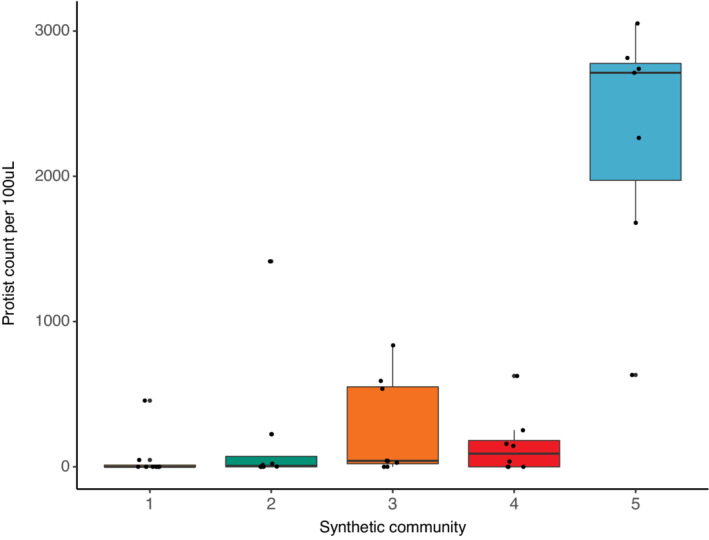
Protist abundance varies depending on a mixture of strains (*F*_[4,34]_ = 29.46, *p* < .001). Any strain combination that contained harmful strains of *Chromobacterium* resulted in poor protozoan growth. Box plot definitions: Center line—median, upper, and lower box limits—upper and lower quartiles, whiskers—1.5× inter‐quartile range, outliers—any points outside the 1.5× inter‐quartile range

Our pairwise interaction experiment demonstrated that bacterial effects on protozoan fitness depend on bacterial strain identity. However, in natural communities, protozoa are unlikely to interact with one bacterial species in isolation. In our second experiment, we found that different community compositions had different effects on protozoan abundance (*F*_[4,34]_ = 29.5, *p* < .001; Figure 2). The negative effects of the “bad” bacterial strains (those that had strong detrimental effects on protozoan fitness) persisted in a community context, where the effects of bad strains outweighed the positive effects of relatively “good” strains (those that have relatively beneficial effects on protozoan fitness). Any combination that included the two bad bacteria from the first experiment (1F and 1I) resulted in low protozoan fitness (Figure 2). This pattern is consistent with bad strains having produced toxins with effects that were not dampened by the presence of other species, rather than the bad strains not supplying sufficient nutrition for the protozoa. It is important to note that our synthetic communities only consisted of six bacterial strains, which is considerably less diverse than a natural community found in a pitcher plant leaf (Gray et al., 2012; Koopman et al., 2010; Koopman & Carstens, 2011), reported to contain approximately 400 bacterial species (Paisie et al., 2014). It is unclear whether the negative effects of the bad bacteria would be dampened in a more diverse bacterial community in a pitcher plant leaf, which has many more microhabitats than a test tube, and where the bad bacterial species would face greater and more diffuse competition for resources with other bacteria.

Simplistically, bacteria are thought to be beneficial to the pitcher plant because they break down insects and release nutrients, while protozoa are thought to be parasitic because they consume bacteria (Baiser et al., 2011; Mouquet et al., 2008). Our results suggest that these categorizations may be more complex since protozoa do not necessarily reduce the abundance of all bacterial taxa evenly. This result also supports previous work in this system and others that demonstrates that protozoa have different prey selection patterns (Gaines et al., 2019; Strom & Loukos, 1998). Furthermore, presumably some bacterial taxa break down prey faster than others, although this has not yet been tested in this system, to our knowledge, nor do we know whether there is a trade‐off between this function and susceptibility to predation. We also do not yet know whether the same taxa‐specific effects of bacteria affect other protozoan consumers in the same way. Future work could expand on these results by doing similar manipulations inside pitcher plant leaves and examining insect decomposition rates or nutrient uptake by the plant. Such information would help us to understand how dynamics among species within the phytotelmic community ultimately affect the plant in which they live.

When *Tetrahymena* protozoa feed, they appear to ingest any bacteria in their immediate surroundings, although they often cluster around structures in the water (e.g., insect parts or pieces of detritus; personal observation). The extent to which bacteria growing on structures in the water differ from those in the water column, or whether good and bad bacteria differ between these environments is unknown and would be an interesting avenue for future work in this system. Protozoa have top‐down effects on bacterial community composition (Canter et al., 2018; Cochran‐Stafira & von Ende, 1998; Holdridge et al., 2016; Paisie et al., 2014; Peterson et al., 2008), and this, combined with the knowledge that different strains affect protozoan fitness, would suggest that the protozoa may engage in a more active form of prey selection than previously thought. There are, however, alternative explanations for the top‐down effects and prey selection patterns of the protozoa on bacterial community composition. For example, some bacterial taxa may have faster growth rates and so can recover more quickly from grazing, or some bacteria may congregate in microniches where protozoa are more abundant, making them more likely to be eaten.

Overall, our results demonstrate that bacterial prey identity affects consumer fitness and that it is vital that future work in these pitcher plant communities account for bottom‐up effects of different bacterial taxa on protozoan growth and fitness, both by taking this into account in models, but also by considering this in empirical studies. For example, Miller and terHorst (2012) found only a weak relationship between bacterial abundance and protozoan abundance across a successional sequence in pitcher plant leaves. However, our work suggests that examining relationships between particular types of bacteria and their effects on the abundance of different protozoa species, and vice versa, could reveal previously unrecognized ecological patterns. Future work could consider how to manipulate or quantify the bacterial community in such a way to tease apart these dynamics. These results open up new avenues of research in pitcher plant microcosms that allow for the study of bottom‐up effects by maintaining lab protozoa aseptically, or with known bacterial taxa, allowing for manipulation of the bacterial community during lab experiments. Alternatively, researchers who either create their own synthetic communities or identify bacterial community members through shotgun sequencing can be aware of which bacterial taxa are present and can account for those effects appropriately, rather than treating the bacterial community as a single unit.

Like other brown food webs, pitcher plant microbial communities are, in practice, often treated as food chains, where the bacteria at the base of the food web are assumed to be functionally redundant. This study demonstrates that this assumption is not valid and that when bottom‐up effects are being examined in this system, it is important to consider the identity of the bacterial community members and their effects on the predators that feed on them. We believe that the results of this study can be further extrapolated to other brown food webs, suggesting that we must move away from treating the foundation of these webs as a black box.

## AUTHOR CONTRIBUTIONS

**Lily Khadempour:** Conceptualization (lead); formal analysis (equal); investigation (equal); methodology (equal); supervision (equal); validation (equal); visualization (lead); writing – original draft (equal); writing – review and editing (equal). **Leslie Rivas Quijano:** Investigation (equal); methodology (equal); validation (equal); writing – original draft (supporting). **Casey P. terHorst:** Conceptualization (supporting); funding acquisition (lead); project administration (lead); supervision (lead); writing – original draft (equal); writing – review and editing (equal).

## Supporting information


Appendix S1

## Data Availability

The data for this study are available in the Zenodo online repository with DOI https://doi.org/10.1101/2021.09.02.458769.
