# Supplementary material for: Prey identity affects fitness of a generalist consumer in a brown food web
Source: Ecol Evol. 2022 Aug 23;12(8):e9207. doi: 10.1002/ece3.9207 (PMC9896622; doi:10.1002/ece3.9207)
Supplement: Supplementary file 1 — Appendix S1 [file ECE3-12-e9207-s001.docx]

**Supplemental Materials**

*Comparison of treatments by biomass*

When initially designing this experiment, we expected that protozoan cell size might respond to different bacterial strains, and that potentially smaller protozoa might reproduce more quickly, so that considering abundance alone might mask the *Tetrahymena* fitness response to the different bacterial strains. Therefore, for the first experiment where we grew individual strains of bacteria with individual genotypes of *Tetrahymena*, we calculated a proxy for biomass. After cells were counted for abundance, we photographed them using ImageJ (National Institutes of Health, http://rsb.info.nih.gov/ij/) and then used the program to calculate cell area for the first 30 individuals observed. We multiplied this number by the abundance of cells per 100μL to get an estimate of total protozoan biomass. Our methods for statistical analysis were the same as for the abundance data.

The results of including cell size did not change the conclusions of the study, but can be seen in Figure S1 and Table S5. While the conclusion, that bacterial strain matters for *Tetrahymena* fitness, did not change, the data became somewhat more opaque. We found that it was beneficial to report abundance only in the main reporting of results. As well, because of the effort required to calculate cell size, we decided not to do this for the second experiment where we mixed bacterial strains.

**Table S1.** Summary of strains isolated, their genera, and their morphotypes.

| Bacterial strain | Genus | Morphotype |
| --- | --- | --- |
| 1H | Serratia | translucent white |
| 1J | Serratia | white |
| 3H | Serratia | translucent white |
| 4E | Serratia | milky shiny white |
| 4L | Serratia | opaque white shiny |
| 4M | Serratia | translucent white |
| 1F | Chromobacterium | dark blue/black |
| 1I | Chromobacterium | black |
| 2G | Chromobacterium | brown |
| 3C | Chromobacterium | matte white |
| 3G | Chromobacterium | light brown ombre |
| 3J | Chromobacterium | dark purple |
| 3M | Chromobacterium | light brown ombre |
| 4B | Chromobacterium | dark purple |
| 4J | Chromobacterium | light, yellowish brown |
| 3F | Burkholderia | white and shiny |
| 3N | Burkholderia | white/round/shiny small |
| 4G | Burkholderia | white |
| 4C | Bacillus | white |
| 2K | Acinetobacter | shiny white |
| 1D | Chryseobacterium | yellow translucent |

**Table S2.** Strain combinations for synthetic bacterial communities.

| Mixture name | Strains included |
| --- | --- |
| Community 1 | 4J, 1F, 1I, 3H, 4C, 3M |
| Community 2 | 4J, 1F, 3H, 3M, 4M, 3F |
| Community 3 | 3J, 4G, 4J, 1F, 4C, 3F |
| Community 4 | 3J, 4G, 2K, 4J, 1F, 1I |
| Community 5 | 3J, 4G, 2K, 3N, 3C, 4L |

**Table S3.** Tukey HSD categories for protist abundance by bacterial strain

| Bacterial strain | Mean protist count per 100μL | Tukey HSD groups |
| --- | --- | --- |
| 3F | 3181.5 | a |
| 2C | 3168.8 | ab |
| 4B | 2901.9 | abc |
| 1H | 2753.1 | abc |
| 3G | 2660.6 | abcd |
| 4E | 2608.0 | abcd |
| 4L | 2533.8 | abcd |
| 1J | 2502.5 | abcd |
| 3N | 2265.4 | abcde |
| 4M | 2233.3 | abcde |
| 3J | 2228.8 | abcde |
| 3H | 2165.9 | abcde |
| 4G | 2073.3 | abcde |
| 2K | 1825.0 | bcdef |
| 2G | 1803.8 | cdef |
| 4C | 1660.0 | cdef |
| 4J | 1397.9 | defg |
| 1D | 1383.0 | defg |
| 3M | 1017.5 | efgh |
| 3C | 680.8 | fgh |
| 1I | 198.9 | gh |
| 1F | 0.0 | h |

**Table S4.** Tukey HSD categories for protist abundance by bacterial strain mixture

| Bacterial strain mixture | Mean protist count per 100μL | Tukey HSD groups |  |
| --- | --- | --- | --- |
| Community 5 | 2271.2 | a |  |
| Community 4 | 259.4 | b |  |
| Community 2 | 209.2 | b |  |
| Community 3 | 152.0 | b | |
| Community 1 | 62.8 | b |  |

**Table S5.** Tukey HSD categories for protist biomass* by bacterial strain

| Bacterial strain | Mean protist abundance | Tukey HSD groups |
| --- | --- | --- |
| 3F | 5.32E+06 | a |
| 2C | 5.24E+06 | a |
| 4B | 4.99E+06 | a |
| 1H | 4.64E+06 | ab |
| 3G | 4.34E+06 | ab |
| 4E | 4.30E+06 | abc |
| 4L | 4.28E+06 | abc |
| 1J | 4.27E+06 | abc |
| 4M | 3.86E+06 | abc |
| 3N | 3.76E+06 | abc |
| 3J | 3.73E+06 | abc |
| 3H | 3.64E+06 | abcd |
| 4G | 3.49E+06 | abcd |
| 2K | 3.13E+06 | abcd |
| 2G | 3.13E+06 | abcd |
| 4C | 2.85E+06 | abcde |
| 4J | 2.42E+06 | bcdef |
| 1D | 2.17E+06 | bcdef |
| 3M | 1.77E+06 | cdef |
| 3C | 1.15E+06 | def |
| 1I | 3.71E+05 | ef |
| 1F | 8.75E-01 | f |

*Biomass is calculated as the abundance of protists per 100μL multiplied by the average area of the protists in the sample.

**Figure S1.** Protist biomass depends on the identity of bacterial strain (F_(21,153)_ = 9.15, P < 0.001). Box plot definitions: center line – median, upper and lower box limits – upper and lower quartiles, whiskers – 1.5 x inter-quartile range, outliers – any points outside the 1.5x inter-quartile range.
